# The Role of Trace Elements in Pseudoexfoliation Syndrome: A Cross-sectional Study

**DOI:** 10.18502/jovr.v16i2.9079

**Published:** 2021-04-29

**Authors:** Mohammad Reza Talebnejad, Ali Azimi, Mohammad Reza Khalili, Aidin Meshksar

**Affiliations:** ^1^Poostchi Ophthalmology Research Center, Department of Ophthalmology, Shiraz University of Medical Sciences, Shiraz, Iran

**Keywords:** Pseudoexfoliation, Trace Element, Zonular Stability

## Abstract

**Purpose:**

Pseudoexfoliation syndrome (PXF) is an age-related condition, characterized by deposition of whitish ﬂake-shaped materials in the anterior segment of the eye. Although it occurs all over the world, a considerable racial variation exists. According to the high frequency of PXF in Iran and the importance of prevention and early treatment, we evaluated the plasma level of iron, zinc, copper, and magnesium in patients with PXF.

**Methods:**

In this study, 83 individuals were enrolled; 40 patients with cataract and PXF as the case group and 43 age- and sex-matched individuals with cataract but without PXF as the control group. The serum levels of the mentioned microelements were compared in two groups.

**Results:**

In the case group, 25 (62.5%) male and 15 (37.5%) female subjects participated. In the control group, the corresponding figures were 22 (51.2%) and 21 (48.8%), respectively. The mean age of the case group was 66.07 ± 9.46 and that for the control group was 66.88 ± 8.04 years. Regarding the case group, the serum levels of iron, zinc, copper, and magnesium were 60.58 ± 21.04, 84.7 ± 14.37, 120.23 ± 14.43, and 2.11 ± 0.23, respectively. These serum levels in the control group were 89.07 ± 26.06, 97.51 ± 17.42, 123.33 ± 19.01, and 2.14 ± 0.16. The serum levels of iron and zinc were significantly lower in the case group than the control group (*P* < 0.0001); however, such a difference was not observed in terms of copper and magnesium serum levels.

**Conclusion:**

Our study demonstrated that the serum iron and zinc levels were lower in PXF patients. Nutritional deficiency may be a cause of zonular weakness in these patients. *Heme* is a cofactor for the enzyme which contributes to the biosynthesis of fibrillin, the major protein in zonular fibers. Therefore, iron can play a substantial role in the biosynthesis of the fibrils and also in the zonular stability.

##  INTRODUCTION

Pseudoexfoliation syndrome (PXF) is an age-related condition which is clinically related with a variety of ocular disorders, including cataract, glaucoma, and increased risk of complications during intraocular surgery.^[1]^ The syndrome is characterized by deposition of whitish ﬂake-shaped fibrillar materials in the intra- and extraocular tissues, more frequently on the anterior lens capsule and can also be detected on the lens zonules, iris surface, corneal endothelium, trabecular meshwork, anterior hyaloid surface and less frequently on the posterior lens capsule and even intraocular lens.^[1,2]^ Due to the accumulation of pseudoexfoliative material in different visceral organs, PXF syndrome is known as a systemic disorder.^[3]^ Most cases can be diagnosed by a careful ophthalmic examination, especially after pupillary dilation.^[2,3]^ Regarding the epidemiology, although PXF occurs all over the world, a considerable racial variation exists. The prevalence of PXF syndrome varies (0.69–23%) with the region and the study design.^[1,2][3][4]^

The etiology of PXF syndrome is not exactly known, but a multifactorial complex model including genetic inheritance combined with environmental factors, such as geographic location, UV exposure, and decreased serum and aqueous levels of antioxidants have been suggested as the contributing factors.^[5,6,7,8,9,10]^

According to the recent studies, oxidative stress plays a key role in the pathogenesis of PXF syndrome and decreased levels of different antioxidants has been investigated in the serum and aqueous samples of patients suffering from PXF syndrome.^[2,3][4][8][9][10]^ Although in a few studies, the serum and aqueous levels of some microelements have been determined in these patients, the possible effect of microelements on the prevalence and/or severity of PXF syndrome has remained controversial.^[11,12,13]^

Based on the previous studies and considering the high frequency of PXF in Iran, especially in rural areas and in malnourished subjects, the authors aimed to design the present study to determine the serum levels of iron, copper, zinc, and magnesium in patients with pseudoexfoliative cataract compared with the control group.

##  METHODS

### Patient Selection

In this cross-sectional study, using a simple random sampling method, 40 patients with visually significant senile nuclear cataract and pseudoexfoliation as the case group and 43 age- and sex-matched individuals with the same type of cataract but without PXF as the control group were enrolled. Patients with secondary types of cataract such as traumatic or metabolic were excluded. All patients were referred to Khalili Hospital, affiliated to Shiraz University of Medical Sciences, Shiraz, Iran for cataract surgery. All experiments were performed according to the Declaration of Helsinki and informed consent was obtained from all individuals. Our local medical ethics committee approved the study. Other exclusion criteria were history of ocular trauma, glaucoma, keratoconus, uveitis, any type of anemia, thyroid dysfunction, diabetes mellitus, Wilson disease, collagen vascular and autoimmune diseases, and also those who received mineral supplements. Patients were assessed for the presence or absence of pseudoexfoliative material on the border of pupil and on the anterior lens capsule and zonules after pupillary dilation.

After at least 8 hr of night fasting, 10 ml blood sample was taken from each patient before cataract surgery. Half of that was used to measure the level of hemoglobin using colorimetric technique and the remaining was centrifuged at 2400 rpm for 15 min to separate the serum. If the hemoglobin level was <14 mg/dl for males or 12 mg/dl for females, the individual was excluded. Serum samples were kept in a freezer at –20°C until analysis.

In this study, colorimetric technique was used for the measurement of Cu, Zn, and Mg levels. The protocol for the measurement of Cu was mixing 1000 lambda (λ) reagent with 50 λ serum. Then, these two solutions were incubated for 10 min at 37°C. Then, colorimetric method was carried out. The protocol for Zn was the same as Cu. For Mg, the 10λ serum was mixed with 1000 λ reagents, and incubation time and temperature were the same as Cu and Zn. Copper, Zn, and Mg levels were measured at 580, 560, and 546 nm wavelengths, respectively. For iron (Fe), auto analyzer colorimeter (Hitachi 912) was used. After mixing 300 λ reagents with 50 λ serum and 20 min incubation at 37°C, the auto analyzer was used. Data were reported in μg/dl for Cu and Zn, and mg/dl for Fe and Mg.

### Statistical Analysis

Data were recorded and analyzed using SPSS Statistics software, version 18 (SPSS Inc., Chicago, IL). Quantitative data were presented as mean ± standard deviation (SD). We used Shapiro–Wilk to check the normal distribution of data. It was shown that Fe and Cu had normal distribution, but for Mg and Zn there was no normal distribution. Independent *t*-test and Mann–Whitney U-test were used to compare the measurements obtained from quantitative variables and Chi-square test were applied for qualitative variable parameter. P-value < 0.05 was considered as statistically significant.

##  RESULTS

Forty patients were enrolled in the case group and 43 patients in the control group. There were no significant differences between the groups in terms of age and sex [Table 1].

**Table 1:** Comparison of the two groups in terms of demographic data and trace element values


**Group**	**Frequency**	**Age${}^{a,d}$**	**Sex${}^{b,e}$**	**Serum Fe(mg/dl)${}^{a,f}$**	**Serum Zn(μg/dl)${}^{a,g}$**	**Serum Cu(μg/dl)${}^{c,h}$**	**Serum Mg(mg/dl)${}^{c,i}$**
Control	43	66.86 ± 8.046	F = 21 (48.8%) M = 22 (51.2%)	89.07 ± 26.063	97.51 ± 17.421	123.33 ± 19.011	2.144 ± 0.1637
Case	40	66.03 ± 9.467	F = 15 (37.5 %) M = 25 (62.5 %)	60.58 ± 21.044	84.70 ± 14.376	120.23 ± 14.437	2.118 ± 0.0379
${}^{a}$Independent *t*-test was applied; ${}^{b}$Chi-square test was applied; ${}^{c}$Mann–Whitney U-test was applied; ${}^{d}$ *P*-value = 0.66; ${}^{e}$ *P*-value = 0.30; ${}^{f}$ *P*-value < 0.001; ${}^{g}$ *P*-value < 0.001; ${}^{h}$P-value = 0.40; ${}^{i}$ *P*-value = 0.55							

As Table 1 shows, the plasma levels of iron and zinc were significantly lower in patients assigned to the case group compared with the control ones (*P*-value < 0.001), however, there was no significant difference between the serum values of Cu and Mg between the two groups (*P* = 0.40, *P* = 0.55, respectively).

##  DISCUSSION

Psuedoexfoliation syndrome (PXF) is a systemic disorder with ophthalmic manifestations.^[14,15,16,17,18]^ The prevalence of PXF increases with age and is characterized by accumulation of fleck-like material in the anterior segment of the eye, especially around the pupillary border and on the surface of the lens. This material is primarily produced by the ciliary body epithelium, the pigmented epithelium of the iris, and also the pre-equatorial lens epithelium. The corneal endothelium, trabecular cells, vascular endothelia, and iris smooth muscle cells have also been suggested to play a role.^[19,20,21]^ PXF material has a complex content which is mainly glycoprotein/proteoglycan composition and also elastic fibers epitopes.^[22,23,24]^ A common gene variant in the Lysyl oxidase homolog 1 (LOXL1 ) enzyme, an enzyme critical for enhancing the tensile strength of collagen and elastin in extracellular matrices, has been found in approximately 90% of the PXF cases.^[6,25,26]^ On the other hand, different environmental factors such as solar radiation, oxidant/antioxidant imbalance, and dietary factors such as high coffee consumption and low dietary folate intake are associated with increased risk of PXF.^[7,8,9,10,27]^ The role of trace elements in the hormonal imbalance has been investigated in previous studies. Zhang et al showed that Cu level increases and Zn level decreases in polycystic ovarian syndrome which is related to hormonal imbalance.^[28]^ Our study showed a reduction in serum level of Zn that can be due to the role of hormonal imbalance in the pathophysiology of PXF. The possible role of microelements on the prevalence or severity of PXF syndrome is a hot topic which has remained controversial based on different studies. Yildirim et al reported that zinc level was decreased in the lens of cataract patients with PXF, and since zinc, copper and magnesium are essential for some metalloenzymes such as superoxide dismutase; there is a possibility of defective antioxidation mechanisms.^[29]^ In another study, decreased level of selenium, which is an essential component of selenoproteins, in aqueous and conjunctiva of patients suffering from PXF, supported the possibility of oxidant/antioxidant impairment in the pathogenesis of PXF syndrome.^[30]^ Cumurcu et al showed that a significant increase in serum total oxidative stress status occurred in PXF.^[31]^

As PXF is considered a systemic disorder, a simultaneous mechanism of local production of the material by anterior segment tissues and systemic accumulation of the material has been proposed. Therefore, measurement of trace element levels in the serum may be helpful. Our investigation showed decreased level of serum iron and zinc in patients with PXF. This results is in contrast with those of the study conducted by Cumurcu et al which showed the elevated level of serum iron in PXF compared with the control group, but such a difference was not observed for zinc.^[11]^ Hohberger et al showed that the aqueous level of Zn was decreased in PXF that is concordant to our results but there was an increase in iron level in PXF and primary open angle glaucoma.^[12]^ Another study performed by Ceylan et al demonstrated that serum level of Zn was not different in PXF and control groups; instead, some other trace elements such as manganese, molybdenum, and mercury were elevated in the serum of patients with PXF and PXF glaucoma.^[13]^ One of the reasons for the difference in the results of our study and some of the mentioned researches might be the distribution of the serum level of iron in our population that is normally distributed, but in the aforementioned studies, this level was not normally distributed among the population. Thus, trace elements may play different roles in different populations according to their distribution. This may support the effect of geographic location in the etiology of PXF syndrome. As microelements may play a role in the synthesis of exfoliative materials, it can also be supposed that the content of these materials might be different in patients affected by PXF syndrome according to the patients' geographical area.

On the other hand, our study has another message, which is the possible role of serum iron deficiency in the weakness of zonular fibers because iron is incorporated in the *heme* structure. There is a *heme*-containing cofactor in the biosynthesis of homocysteine that has a regulatory role and its absence leads to an increase in the homocysteine level.^[32,33]^ Hubmacher et al showed that homocysteine can alter the structure and/or function of the microfibrils which contain fibrillin and cause zonular weakness and/or laxity.^[34]^ Previous studies showed that the homocysteine plasma level is increased in patients with PXF and the serum level of homocysteine is correlated with the severity of PXF.^[35,36,37,38]^ Homocysteine may be a correctable risk factor in the pathogenesis of PXF and elevated homocysteine level has been shown to be reversible by consumption of vitamins.^[23]^ The results of our study can demonstrate the possible role of iron deficiency in the pathogenesis of zonular fiber weakness in PXF through the homocysteine pathway as iron is a part of the cofactor in the homocysteine metabolism.

In conclusion, although the accumulation of exfoliative materials in the zonula is considered as the core mechanism of zonular weakness in PXF, our investigation also suggests a minor pathway. This is due to iron deficiency and occurs through the homocysteine pathway. Therefore, further researches are needed to evaluate whether iron supplementation has any effect on the zonular stability.

Our study had some limitations. The first is that our sampling was from a single center; it may be helpful to have different groups of patients with PXF from different geographical locations for future researches. In addition, a larger sample size can be helpful for future studies.

##  Financial Support and Sponsorship

No financial support was received for this submission.

##  Conflicts of Interest

The authors declare there are no conflict of interest.
